# Supplementary material for: Reconciling Mining with the Conservation of Cave Biodiversity: A Quantitative Baseline to Help Establish Conservation Priorities
Source: PLoS One. 2016 Dec 20;11(12):e0168348. doi: 10.1371/journal.pone.0168348 (PMC5173368; doi:10.1371/journal.pone.0168348)
Supplement: S1 Dataset — (ZIP) [file pone.0168348.s002.zip › Taxa/Serra Norte/SN_2013/N4WS/Lista N4WS-13.pdf]

CAVIDADE N4WS-0013

Classe	Ordem	Fam/Outros	Gên/Outros	Espécie	S-U	U-S	Zon.
Annelida	Hirudinea			sp.1		1	P
Annelida	Oligochaeta			sp.		4	
Arachnida	Acari	(Parasitengonina)		sp.2		X	
Arachnida	Acari	Argasidae	*Ornithodoros*	sp.1	X	X	
Arachnida	Acari	Ixodidae	*Amblyomma*	*cajennense*	X		
Arachnida	Acari	Mesostigmata		jovem	X		
Arachnida	Acari	Rhagidiidae		sp.2		X	P
Arachnida	Acari	Trombiculidae		sp.	X		
Arachnida	Amblypygi	Charinidae	*Charinus*	sp.1		2	E
Arachnida	Araneae	Araneidae		jovem	X		
Arachnida	Araneae	Ctenidae		jovem		1	P
Arachnida	Araneae	Dipluridae		jovem	1		
Arachnida	Araneae	Drymusidae		jovem		X	E
Arachnida	Araneae	Ochyroceratidae	*Ochyrocera*	sp.1	X	X	E/P
Arachnida	Araneae	Ochyroceratidae	*Speocera*	sp.	X		
Arachnida	Araneae	Pholcidae	*Leptopholcus*	sp.1		X	E
Arachnida	Araneae	Prodidomidae		sp.	X		
Arachnida	Araneae	Salticidae		sp.	X		
Arachnida	Araneae	Scytodidae	*Scytodes*	jovem	1	2	E/P
Arachnida	Araneae	Segestriidae		jovem	X		
Arachnida	Araneae	Theraphosidae		sp.	X		
Arachnida	Araneae	Theridiidae		jovem		X	E
Arachnida	Araneae	Theridiosomatidae	*Plato*	sp.1	X	X	E
Arachnida	Palpigradi			jovem	X	X	E
Arachnida	Pseudoscorpiones	Chernetidae	*Spelaeochernes*	sp.1		X	E/P
Arachnida	Pseudoscorpiones	Chthoniidae	*Pseudochthonius*	sp.1	X	X	E/P
Chilopoda	Geophilomorpha	Ballophilidae	*Ityphilus*	sp.1		1	P
Chilopoda	Scolopendromorpha	Scolopocryptopidae	*Dinocryptops*	*miersii*	X		
Diplopoda	Polydesmida	Chelodesmidae		sp.	X	1	
Diplopoda	Polydesmida	Pyrgodesmidae		sp.1		X	P
Diplopoda	Polyxenida	Polyxenidae		jovem	X	X	P
Diplopoda	Siphonophorida	Siphonophoridae		jovem		X	P
Entognatha	Collembola	Cyphoderidae		sp.1		X	P
Entognatha	Collembola	Paronellidae		sp.4		X	E/P
Entognatha	Collembola	Sminthuroidea		sp.	X		
Entognatha	Diplura	Campodeidae		sp.1	X	X	E
Entognatha	Diplura	Japygidae		sp.1		X	P
Insecta	Blattodea			jovem	X	3	E/P
Insecta	Coleoptera	Carabidae		sp.	X		
Insecta	Coleoptera	Dytiscidae	*Copelatus*	*cessaima*	X	X	P
Insecta	Coleoptera	Dytiscidae		sp.14		X	P
Insecta	Coleoptera	Lampyridae		jovem	X		
Insecta	Coleoptera	Staphylinidae		jovem	X		
Insecta	Coleoptera	Tenebrionidae	Alleculinae	jovem	X		
Insecta	Diptera	Cecidomyiidae	Cecidomyiinae	sp.	X	X	E
Insecta	Diptera	Psychodidae	Phlebotominae	sp.		X	E/P
Insecta	Heteroptera	Cydnidae		jovem	X		
Insecta	Heteroptera	Reduviidae	*Zelurus*	sp.	X		
Insecta	Homoptera	Cixiidae		jovem	X	X	E

Insecta	Hymenoptera	Formicidae	*Camponotus*	*atriceps*	X		
Insecta	Hymenoptera	Formicidae	*Camponotus*	sp.2		X	P
Insecta	Hymenoptera	Formicidae	*Carebara*	sp.1	X		
Insecta	Hymenoptera	Formicidae	*Eciton*	sp.1		X	E
Insecta	Hymenoptera	Formicidae	*Hypoponera*	sp.2		X	E
Insecta	Hymenoptera	Formicidae	*Pachycondyla*	*constricta*	X		
Insecta	Hymenoptera	Formicidae	*Pachycondyla*	sp.1		X	E
Insecta	Hymenoptera	Formicidae	*Paratrechina*	sp.	X		
Insecta	Hymenoptera	Formicidae	*Pheidole*	sp.	X		
Insecta	Hymenoptera	Formicidae	*Pheidole*	sp.2		X	P
Insecta	Hymenoptera	Formicidae	*Solenopsis*	sp.1		X	E
Insecta	Hymenoptera	Scelionidae		sp.	X		
Insecta	Hymenoptera	Sphecidae		sp.	X		
Insecta	Isoptera	Termitidae	*Nasutitermes*	sp.	X	X	E/P
Insecta	Lepidoptera	Noctuoidea		sp.	X		
Insecta	Lepidoptera	Tineoidea		sp.	X	X	P
Insecta	Orthoptera	Phalangopsidae	*Phalangopsis*	sp.	X	42	P
Insecta	Psocoptera	Epipsocidae	*Epipsocus*	sp.1	*X*	X	E
Insecta	Thysanura	Nicoletiidae		sp.1		X	P
Malacostraca	Isopoda	Armadillidae		sp.1	X	X	P
Malacostraca	Isopoda	Philosciidae		sp.1	X	X	P
Malacostraca	Isopoda	Scleropactidae	*Circoniscus*	*buckupi*	X		
Symphyla		Scutigerellidae	*Hanseniella*	sp.	X		
Turbellaria				sp.	X		
Amphibia	Anura			sp.		6	E/P
Mammalia	Chiroptera	Emballonuridae	*Peropteryx*	sp.	3	5	P
